# Physico-Chemical and Rheological Trait-Based Identification of Indian Wheat Varieties Suitable for Different End-Uses †

**DOI:** 10.3390/foods13071125

**Published:** 2024-04-08

**Authors:** Sumit Kumar Singh, Shaily Singhal, Praful Jaiswal, Umer Basu, Anant Narayan Sahi, Anju Mahendru Singh

**Affiliations:** 1 Grain Quality Laboratory, Division of Genetics, ICAR-Indian Agricultural Research Institute, New Delhi 110012, India; sumitbiosoft@gmail.com (S.K.S.); shailysinghal11@gmail.com (S.S.); 2 Amity Institute of Biotechnology, Amity University, Noida 201313, India; jaiswalpraful1987@gmail.com (P.J.); ansahi@amity.edu (A.N.S.); 3 College of Plant Protection, Northwest A & F University, Yangling 712100, China; basuumar1608@gmail.com; 4 Key Laboratory of Integrated Pest Management on Crops in Northwestern Loess Plateau, Ministry of Agriculture and Rural Affairs, Yangling 712100, China; 5 Key Laboratory of Plant Protection Resources & Pest Management, Ministry of Education, Yangling 712100, China; 6 Division of Germplasm Conservation, ICAR-National Bureau of Plant Genetic Resources, New Delhi 110012, India

**Keywords:** diversity, dough, genotype, end-use, traits, glu-1 score, quality, rheological, wheat

## Abstract

India has increased its wheat production phenomenally in the last two decades and it now has a buffer stock of 9.7 million tonnes. However, despite the release of several wheat cultivars, the end-use quality traits of Indian wheat varieties have not been explored in-depth to determine the increasing demand of the domestic processing industry as well as export. In this study, 55 wheat genotypes including 47 released varieties, and 8 genetic stocks were grown along with 10 Australian varieties grown during cropping seasons: 2019–2020 and 2020–2021 and diversity in different physiochemical and rheological traits was evaluated. They showed considerable diversity in all the quality traits studied. However, very few genotypes could be found suitable for any one end-use. Five genotypes were found to possess four to five traits for superior bread-making quality. Two varieties and three advanced breeding lines had up to four good chapati quality traits. None of the released varieties investigated had suitable traits for biscuit making; however, two breeding lines possessed requisite quality traits suitable for biscuit making. It is, therefore, concluded that systematic breeding efforts are required to develop genotypes that bring together the most important quality traits in a single genotype to be suitable for domestic industry as well as for export.

## 1. Introduction

Wheat (*Triticum aestivum* L.) stands as the second most cultivated grain crop globally in terms of acreage and production volume. In the marketing year of 2023–2024, the global production volume of wheat reached nearly 784.9 million metric tons [1]. India is the second-largest producer and consumer of wheat globally, with China leading in production. China produced around 135 million metric tons, while India’s production stood at approximately 112 million metric tons during the same period (2023–2024). Among grains, wheat grain/flour stands out for its versatility in producing various end products. However, efforts to understand the processing quality of different wheat varieties in India have been limited. The end-use characteristics of a wheat genotype are significantly influenced by its texture, protein content, and starch characteristics. Indian wheat varieties are notably known for their hard grain texture, which affects milling properties and flour quality. The hardness of the grain determines the milling force required, with hard grains demanding greater force and resulting in more damaged starch granules and increased water absorption capacity of the flour/dough [2]. Research studies have highlighted the importance of endosperm hardness in influencing milling properties, flour yield, starch damage, water absorption, dough consistency, and development time [3,4].

In grain endosperm, gluten serves as the primary storage protein, consisting of glutenins and gliadins. When mixed with water, these proteins interact to form a complex network crucial for dough development [5]. Glutenin, particularly high-molecular-weight glutenin subunits (HMW-GSs), plays a vital role in providing stability and elasticity to the gluten structure [6]. Encoded by genes on chromosome 1A, 1B, and 1D, HMW-GSs exhibit allelic variation influencing gluten properties. The cumulative Glu-score, derived from these alleles, is a key predictor of flour quality [7,8]. Additionally, low-molecular-weight glutenin subunits (LMW-GSs), encoded by Glu-3 loci, contribute to gluten viscoelasticity and are closely linked to gliadin genes on chromosome 1A. Understanding the genetic regulation of these gluten components is essential for improving wheat quality [9,10].

The baking industry adopts various tests to assess the rheological characteristics of wheat flour, predicting its suitability for specific applications without conducting actual baking tests [11,12]. Different wheat flours are recommended based on properties like protein percentage, sedimentation value, damaged starch percent, and gluten strength for various products such as bread, biscuits, and chapati [13,14]. In many wheat exporting countries, wheat is classified into different classes based on its traits in most exporting nations, but such classification is lacking in India. Adopting international standards for evaluating wheat quality is crucial for industry adoption and further quality improvement. In this study, advanced breeding lines and released varieties have been evaluated using internationally accepted methods to ascertain their quality traits and utility for different end products, highlighting potential donors for enhancing end-use quality.

## 2. Materials and Methods

### 2.1. Plant Materials

In the present investigation, a total of sixty-five wheat genotypes were used for study. These included ten advanced breeding lines, forty-five bread wheat varieties that had been released for various agro-climatic zones in India, and ten Australian wheat varieties (Table S1). The experimental trials were conducted with a randomized complete block (RBD) design with two replications at experimental fields of the Indian Agricultural Research Institute (IARI), New Delhi, India, during two consecutive cropping seasons: 2019–2020 and 2020–2021. Each experimental plot comprised two rows, each stretching 3 m in length, separated by a 10 cm spacing between rows, making a sub-plot size of 0.3 m^2^, and the total area of the experimental field amounted to 39 m^2^.

### 2.2. Protein Isolation and SDS-PAGE

An HMW-GS protein from each wheat genotype was isolated from flour of five seeds using the Zhen and Mares method [15]. Flour was mixed with 60% ethanol (*v*/*v*) for 30 min to wash out gliadins. The mixture was centrifuged at 5000 rpm for 10 min, and the supernatant was discarded. The pellet was treated with an extraction buffer of DTT, SDS, 1 M Tris-HCl (pH 6.8). The mixture was vortexed for 30 min and centrifuged at 10,000 rpm for 10 min. Supernatant containing glutenin protein was collected and SDS–polyacrylamide gel electrophoresis (SDS-PAGE) analysis was performed [15]. SDS-PAGE gel was prepared using 10% gradient separation gel (pH 8.5) and a 4% stacking gel (pH 6.8); gel was placed in an electrophoresis tank filled with a running gel buffer of Tris, glycine, and SDS (pH of 8.3), and protein samples were loaded into the wells after adding protein loading dye. Electrophoresis was carried out at a constant current of 40 mA for 4 h, and the gels were stained overnight in solution of 0.1% *w*/*v* Commissive brilliant blue R-250, 50% *w*/*v* methanol, and 10% *w*/*v* trichloro acetic acid. After staining, the gels were rinsed with distilled water and washed on a horizontal shaker until the excess dye was removed. Protein bands on the gel were identified and scored according to standard nomenclature [16], using reference cultivars with known HMW-GSs. Glu-1 scores of the studied lines were determined based on the numeric scale developed by Payne et al. for HMW-GS alleles [7].

### 2.3. Quality Analysis

The evaluation of 17 different traits was conducted using internationally accepted methods of the American Association of Cereal Chemists (AACC, 2000) [17]. The traits included grain hardness index (GHI), thousand kernel weight (TKW), and grain diameter (GD) and were evaluated using the Single Kernel Characterization System (SKCS) using Perten Instruments4100, Sweden, as per the AACC method 55-31.01. The test weight (TW) was evaluated (in kg/hL) by an instrument developed at ICAR-IIWBR as per AACC 55-10.01. The grain was milled using the two-lane Quadrumat Senior mill of Brabender Instruments, Germany. Total bran (TB), fine bran (FB), and flour recovery (FR) were calculated following the AACC method No. 26-50.01. Protein percent (P%) was estimated using the Foss NIRS DS2500 instrument. SDS–sedimentation Value (SDS-SV) was estimated through the AACC method 56-70. Wet gluten (WG), dry gluten (DG), and gluten index (GI) were evaluated using the Glutomatic 2200 of Perten Instruments, Sweden, as per AACC 38-12.02. Water absorption (WA), dough development time (DDT), dough stability (STAB), degree of softening (DOS), and farinograph quality number (FQN) were evaluated using the Farinograph^®^-E from Brabender, Germany, as per AACC method 54-21.02.

### 2.4. Statistical Analysis

Statistical analyses were conducted to elucidate relationships among traits studied across various genotypes. Pearson’s correlation coefficient (r) was employed to assess associations between traits within the studied genotypes, utilizing mean values derived from four replications (two each in 2019 and 2020). This analysis was performed using SPSS 25. Principal Component Analysis (PCA) was used to characterize variability within subsets of variables and explore relationships among traits across genotypes, using XLSTAT statistical package (Version 2014.0.3). Scatter Plot Analysis (SPA) was used to visually represent relationships among numerical variables across genotypes. Agglomerative Hierarchical Clustering (AHC) analysis was conducted to evaluate pairwise genetic similarity among genotypes, based on the Jaccard similarity coefficient to compute a similarity matrix. AHC analysis was carried out using the XLSTAT statistical package (Version 2014.0.3). These statistical methods collectively provided insights into trait relationships and genetic similarity among the studied genotypes.

## 3. Results

### 3.1. Quality Trait Variation and Their Correlations

Each variety is listed along with its respective Glu-A1, Glu-B1, and Glu-D1 subunit compositions, as well as its Glu-1 score (Table S1). The Glu-1 score exhibited a range from 4 to 10 across the studied genotypes. Varieties like Annuello, Barham, and Baxter displayed diverse compositions, with different combinations of gluten subunits contributing to their Glu-1 scores. These subunit compositions play a crucial role in determining the technological properties and end-use quality of wheat varieties (Table 1).

The analysis of 65 wheat genotypes revealed significant variability across 19 distinct quality traits. The grain hardness index exhibited a broad range from 16.087 to 94.21, showcasing considerable diversity among varieties studied. The total bran content varied between 19.62 and 34.04, with fine bran yield ranging from 2.10 to 10.63. Flour recovery after milling displayed a variance of 8.04, ranging from 61.36% to 75.12%, indicating a wide range of milling outcomes influenced by grain texture. The gluten index ranged from 10.169 to 100, reflecting a significant variation in gluten content among varieties also. The protein content ranged from 8.7% to 19.8%, offering potential for diverse end products. The wet gluten content spanned from 21.75% to 43.2% (Table 2), with dry gluten yield ranging from 7.2% to 18.8%. Significantly, around 60% of the genotypes exhibited dry gluten exceeding 10%. SDS–sedimentation value showed substantial variability, ranging from 26 to 68 mL, indicating genetic influences on sedimentation traits. Water absorption ranged from 52.5% to 70.3%, while dough development time ranged from 0.7 to 20 min. The Farinograph quality number ranged from 9 to 200, underscoring the diverse dough quality across genotypes (Table 2). These findings highlight the extensive variability and potential applications of the studied wheat varieties.

Pearson’s correlation analysis conducted on the studied wheat genotypes revealed several significant associations among quality traits (Table 3). Significantly, a +ive correlation was observed between Glu-1 scores, Glu index (GI), and farinograph quality number in baking. The grain hardness index exhibited significant +ive associations with fine bran, dough development time, dough stability, and farinograph quality number (*p* ≤ 0.01). However, grain hardness showed significant −ive correlations with total bran yield and degree of softening at a 99% level of significance. Fine bran and flour recovery also displayed significantly +ive associations with grain hardness index. Flour recovery also exhibited positive correlation with SDS–sedimentation value and −ive correlation with water absorption. A strong +ive correlation was observed between fine bran and water absorption. Furthermore, GI showed +ive correlations with Glu-1 scores, SDS–sedimentation value, farinograph quality number, dough development time, dough stability, and water absorption. Protein percent also showed +ive correlations with SDS–sedimentation value, dry gluten, and wet gluten. SDS–sedimentation value was found to be positively correlated with dry gluten, wet gluten, and Glu index but negatively correlated with thousand kernel weight, grain diameter, and test weight. Similarly, farinograph quality number showed +ive correlations with Glu-1 scores, grain hardness index, SDS–sedimentation value, Glu index, water absorption, dough development time, and dough stability but showed a strong −ive correlation with degree of softening. Additionally, dough development time exhibited significant +ive correlation with degree of softening. These findings contribute to a comprehensive understanding of wheat quality traits.

### 3.2. Principal Component Analysis (PCA) of Quality Traits

**Figure 1:**
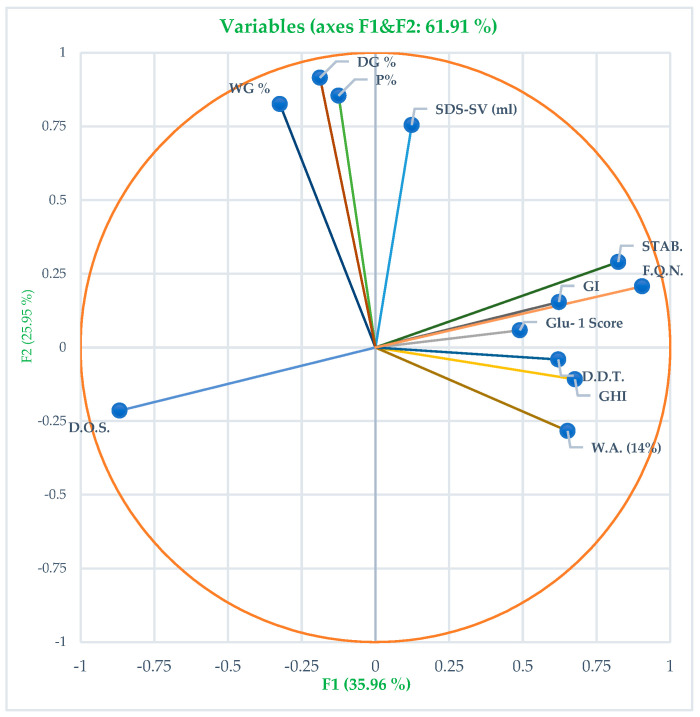
Principal component analysis (PCA) biplot of the measured traits; length of arrows indicates the relative size of contribution of the trait in the PCA.

PCA showed that the first two components captured 61.91% of the differences among the wheat varieties. These components, labeled PC1 and PC2, contributed to the overall variation in the traits (Figure 1). The genotype plot and biplot graph of this analysis are shown in Supplementary Figure S1 and Figure S2, respectively. The traits wet gluten, dry gluten, protein (%), and SDS–sedimentation value fall into the first cluster, among which wet gluten and protein (%) depicted the strongest correlation while wet gluten and SDS–sedimentation value showed the weakest correlation. The plot suggests that wet gluten, dry gluten, and protein percentage can predict high SDS–sedimentation value reliably. On the other hand, traits like dough stability, Farinograph quality number, gluten index, and Glu-1 score tend to cluster together on the right side, showing strong correlations between dough stability and gluten index, as well as Farinograph quality number. Conversely, the weakest correlation was found between degree of softening and Glu-1 score. This means that Farinograph quality number and gluten index are likely to predict the Glu-1 score and vice versa. Since Farinograph data might not be readily available to breeders, analyzing high-molecular-weight gluten can serve as a useful proxy for assessing dough stability and flour quality. Additionally, the loading positions of protein percentage, degree of softening, and Farinograph quality number with dough stability are at right angles, suggesting no direct correlation between them. Therefore, wheat varieties with high protein percentages may not necessarily exhibit high stability or Farinograph quality. Dough development time, grain hardness index, and water absorption traits showed significant correlations, with dough development time and grain hardness index having the strongest correlation, while dough development time and water absorption showed a +ive correlation. Degree of softening, located in the third quadrant, exhibited a negative correlation with dough stability, Farinograph quality number, Glu-1 score, and gluten index.

### 3.3. Dendrogram Construction

**Figure 2:**
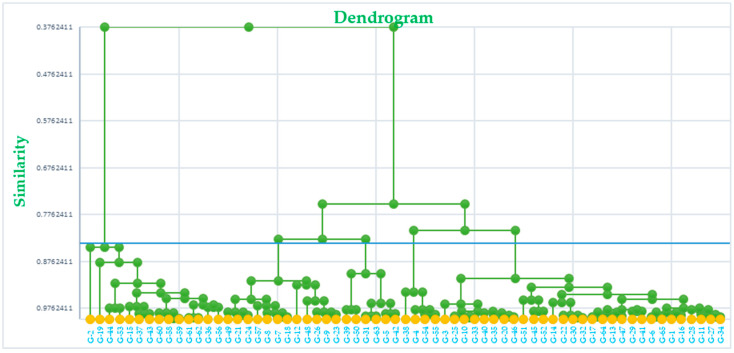
Dendrogram showing the diversity among wheat genotypes.

Dendrogram (Figure 2) was constructed using AHC analysis, revealing the presence of five main clusters based on the similarity matrix. These clusters categorized all genotypes into distinct groups according to their rheological and physiochemical properties. Cluster-I comprised twelve genotypes, including eight Australian varieties (Annuello, Barham, Baxter, Binnu, Datatine, Drysdale, Gladius, Janz), and four Indian genotypes (HPW 311, HQW 2, QBP 13-10, WL 711). Clusters II, III, and IV consisted of 14, 22, and 6 Indian genotypes, respectively, without any Australian varieties. In contrast, Cluster-V included two Australian and nine Indian genotypes.

### 3.4. Scatter Plots

**Figure 3:**
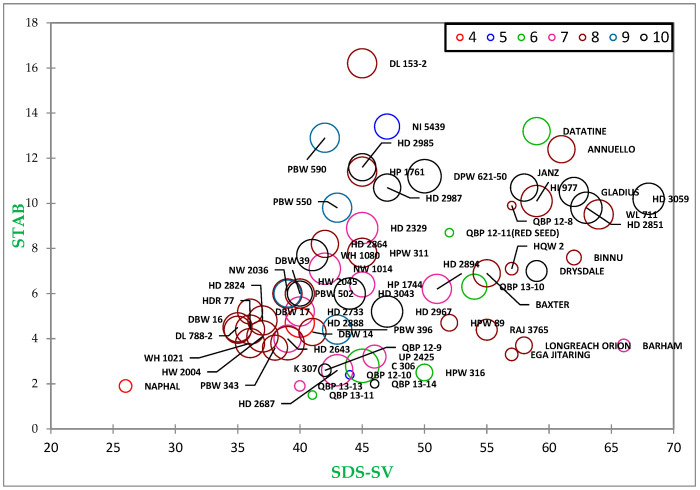
Scattered plot analysis (SPA) showing the diversity among wheat genotypes with STAB, SDS-SV, circle size for respective hardness (higher hardness reflected bigger circle size), and Glu-1 scores; Glu-1 score labelled in different color (details are on top right of graph).

The first scatter plot (Figure 3) was created using degree of softening and SDS–sedimentation value, with Glu-1 scores and grain hardness (represented by the size of the circle). The second plot was constructed using dough stability (STAB) and SDS–sedimentation value with Glu-1 scores. In the SPA graph, the SDS–sedimentation value of the studied genotypes was plotted on the *x*-axis, while DOS was plotted on the *y*-axis. The grain hardness index (GHI) was depicted with the size of the circle in the graph, where larger circle sizes represented higher GHI values. The Glu-1 score of the germplasm was represented with different colors, with details placed in the top right corner of the graph. This SPA construct aids in understanding the clustering of genotypes and their comparative closeness concerning the used traits. It revealed a cluster of Indian genotypes with an SDS-SV ranging from 35 to 45 and STAB from 3 to 11.5.

## 4. Discussion

### 4.1. Variability of Traits among Genotypes

The wheat varieties under study demonstrated a broad spectrum of quality traits, with the Glu-1 score serving as a key indicator of flour gluten strength, also proposed by Payne et al. [16]. These scores are influenced by the presence of HMW-GS in genotypes, with higher scores preferred for bread-making flour. In our study, most genotypes exhibited Glu-1 scores of 8 (41.5%).

Grain texture and hardness are important factors in global grain trade and end-use suitability assessments. Following the classification proposed by Morris et al. [18], based on the grain hardness index obtained from the SKCS, genotypes were categorized into hard, medium hard, medium soft, and soft grain types. Among the genotypes studied, Naphal and advanced breeding lines of the QBP series were identified as having soft grains, while others fell into the medium hard and hard grained categories. Among Australian varieties, Barham, Binnu, EJA Jitaring, and Longreach Orion were classified as soft wheat, while others were categorized as hard grain.

Protein content is another crucial parameter influencing both grain trade and end-product suitability, with its inheritance being highly quantitative and greatly influenced by environmental factors. The P (%) in our study exhibited wide diversity suitable for various end products. Additionally, SDS-SV is used in the industry to predict flour quality, with higher values indicating stronger flour. The majority of genotypes studied were identified as medium strong gluten types. Gluten index (GI) elucidates the elasticity grade and extensibility of flour, with studies indicating a good correlation between GI and SDS-SV, suggesting their capability to assess comparable gluten strength characteristics [19]. Farinograph water absorption (WA) is primarily influenced by the endosperm texture and gluten content. DDT serves as a measure of protein quality, with stronger flours typically requiring longer development times compared to weaker flours [13].

### 4.2. Correlation among Traits

Our study highlighted numerous significant associations between genotypes and rheological traits, emphasizing the key role of kernel shape and size in determining test weight and serving as an approximate indicator of flour recovery [20,21]. Milling quality stands out as a crucial determinant in wheat trade, heavily reliant on grain size and texture. We observed a correlation between Grain hardness index and flour recovery, consistent with findings reported by Katyal et al. [22]. Furthermore, yield components such as test weight, thousand kernel weight, and grain diameter exhibited a −ive correlation with protein and its components (P%, DG, WG, and SDS-SV), aligning with earlier reports [20,21].

Previous studies have highlighted a correlation between Farinograph stability time and flour strength, emphasizing its significance as a key predictor of gluten strength. Notably, significant correlations between bread volume and STAB have also been reported [23]. Typical bread flour characteristics include higher water absorption (≥60%), a dough development time of ≥3 min, and STAB of ≥8 min [24]. In our sample set of 65 genotypes, 26.66% of genotypes met at least two of the criteria for bread flour quality, while several genotypes displayed characteristics indicative of soft grain, suggesting decreased tolerance to mixing. This variability underscores the broad range of weak to strong gluten suitable for various end-use products.

Wheat bran, a by-product of the milling process, typically constitutes 14–19% of the total grain weight [25]. In our study, we observed a positive correlation between flour bran and flour recovery with grain hardness, along with +ive correlations between flour bran and water absorption. These findings are consistent with earlier studies [24,25], which reported that an increase in bran proportion leads to higher flour protein (%). Furthermore, our investigation confirmed +ive correlations between protein (%) and quality attributes SDS-SV and wet and dry gluten in line with previous research [26,27]. SDS-SV, linked to the swelling of gluten strands, emerged as an indicator of genotype-specific gluten strength, supported by other studies demonstrating a close +ive association between gluten strength and SDS-SV [28,29]. The PCA study highlighted the potential of certain traits like wet gluten and protein percentage as predictors for flour quality. Additionally, it emphasized the importance of high-molecular-weight analysis in assessing dough stability and quality. Genotypes with high protein content did not always exhibit high stability. The findings underscored correlations between various traits, providing valuable insights for wheat breeders.

### 4.3. Genotypes Identified for End-Use Based on Scatter Plot Analysis

Quality assessment in wheat involves a multifaceted approach, as superior end-use suitability necessitates the presence of multiple quality traits within specific ranges. While various indirect tests have been devised to assess wheat technological quality, none have proven entirely satisfactory. Thus, in our study, a combined interpretation of these tests using Scatter Plot Analysis proved valuable in determining the flour suitability for different end-uses. An ideal bread-making flour is characterized by high protein content, high sedimentation value, high water absorption, high stability time, high gluten index, and low degree of softening [30]. Higher protein content enhances water absorption, necessitating a longer mixing time for optimal dough consistency [12]. Conversely, low-protein content (8–10%), low water absorption, and low dough stability are deemed suitable for biscuit making [31]. Previous studies have highlighted the suitability of hard wheat (high GHI) for bread and chapati making, while soft wheat (low GHI) varieties are preferred for biscuit making [32,33].

The data analysis revealed limited diversity in the studied wheat lines regarding quality traits. Most Indian genotypes clustered within a specific range of SDS-SV and STAB, indicating a narrower variability. Some Australian and Indian lines exhibited traits suitable for bread making, while others showed potential for biscuit making. Varieties like UP2425 and HD2687 demonstrated traits similar to C306, making them potential alternatives for chapati production. Breeding lines like QBP 12-8 and QBP 12-10 showed promise for biscuit making, albeit with certain limitations. Notably, the Indian variety Naphal could be valuable for enhancing biscuit making quality. While the optimal quality traits for chapati making have not been extensively researched, high-quality chapati typically requires flour with high protein content, significantly damaged starch, and notable water absorption capacity [34]. Recent studies have suggested that whole meal flours with lower to moderate dough stability yield better chapatis, with a preference for medium–strong dough [35]. Furthermore, a chapati’s tearing force is influenced by protein/gluten content and dough development time [31]. Harisha (2023) conducted an in-depth analysis comparing the quality traits of the landmark Indian wheat variety C306, renowned for its chapati quality, with 40 other wheat varieties of India. Varieties with hard to very hard grain, low to moderate protein, low sedimentation value, and low to moderate dough stability demonstrated outstanding chapati quality [36].

## 5. Conclusions

The extensive analysis conducted in our study highlights the considerable variability present among Indian wheat varieties across various quality traits. However, when considering the combination of traits, only a few genotypes exhibit the desirable characteristics sought after by end-users. Currently, many wheat varieties released through national breeding programs prioritize yield and disease resistance, lacking targeted quality traits. Consequently, millers often need to adjust milling conditions to optimize flour suitability for specific applications. Our research findings identify genotypes suitable for different end-use categories by comprehensively analyzing multiple traits. The demand for wheat-based ready to eat foods is experiencing significant growth, expanding at an annual rate of 14%, and is projected to have a market worth of $25 billion. To meet this demand, targeted breeding programs focusing on specific traits are crucial. Both the milling and baking industry and research organizations should collaborate to invest in quality breeding programs and enhance the capacity of human resources. This collaborative effort will pave the way for the development of wheat varieties tailored to meet the evolving demands of the food industry, ensuring superior end products for consumers.

## Figures and Tables

**Table 1:** Glu-1 scores frequency and percentage in wheat genotypes.

Glu-1 Scores	Frequency	Percent
4	2	3.1
5	2	3.1
6	6	9.2
7	10	15.4
8	27	41.5
9	4	6.2
10	14	21.5
Total	65	100

**Table 2:** The mean values of quality parameters of grain and flour of different wheat genotypes.

Parameters	Mean	Std. Error ± Mean	Std. Dev.	Variance	Range	Minimum	Maximum
Glu-1 scores	7.94	0.18	1.49	2.24	6	4	10
GHI	66.90	2.88	9.23	539.94	78.12	16.08	94.21
TKW (gm)	35.65	0.80	6.52	42.63	32.74	21.21	53.96
GD (mm)	2.72	0.03	0.25	0.06	1.22	2.13	3.35
TW (kg/hL)	75.72	0.54	4.36	19.07	23.21	58.53	81.74
TB (%)	26.21	0.31	1.51	6.64	14.41	19.62	34.04
FB (%)	6.24	0.30	1.67	6.11	8.52	2.10	10.63
FR (%)	67.54	0.35	2.83	8.04	13.76	61.36	75.12
SDS-SV (mL)	47.03	1.14	3.19	84.46	42	26	68
P (%)	13.39	0.25	1.02	4.10	11.10	8.77	19.88
WG (%)	32.03	0.65	5.26	27.71	21.44	21.75	43.20
DG (%)	11.15	0.28	2.27	5.15	11.62	7.22	18.84
GI	67.18	2.94	9.78	65.59	89.83	10.16	100
WA (14%)	61.66	0.49	3.95	15.66	17.8	52.5	70.3
DDT (min)	5.58	0.65	5.24	17.51	19.3	0.7	20.0
STAB (min)	6.60	0.42	1.42	11.74	14.7	1.5	16.2
DOS (FU)	58.69	5.80	16.80	190.27	182	1	182
FQN	84.32	5.73	26.22	136.37	191	9	200

**Table 3:** Pearson’s correlation coefficients between different quality traits of wheat genotypes.

	Glu-1 Score	GHI	TKW (gm)	GD (mm)	TW kg/hL	TB (%)	FB (%)	FR (%)	SDS-SV (mL)	P (%)	WG (%)	DG (%)	GI	WA (14%)	DDT	STAB	DOS	FQN
Glu-1 Score	1																	
GHI	0.263 *	1																
TKW (gm)	−0.091	−0.022	1															
GD (mm)	−0.11	0.082	0.962 **	1														
TW kg/hL	−0.003	0.276 *	0.724 **	0.719 **	1													
TB (%)	−0.264 *	−0.410 **	−0.505 **	−0.537 **	−0.557 **	1												
FB (%)	0.112	0.683 **	0.393 **	0.464 **	0.458 **	−0.369 **	1											
FR (%)	0.143	0.322	0.477 **	0.084	0.417 **	−0.587 **	−0.536 **	1										
SDS-SV (mL)	0.208	−0.177	−0.448 **	−0.491 **	−0.594 **	0.13	−0.549 **	0.360 **	1									
P (%)	−0.038	−0.059	−0.661 **	−0.610 **	−0.572 **	0.376 **	−0.399 **	0.006	0.434 **	1								
WG (%)	−0.19	−0.114	−0.660 **	−0.631 **	−0.585 **	0.411 **	−0.493 **	0.056	0.393 **	0.779 **	1							
DG (%)	−0.056	−0.113	−0.631 **	−0.619 **	−0.664 **	0.316 *	−0.499 **	0.148	0.547 **	0.812 **	0.891 **	1						
GI	0.408 **	0.179	0.024	−0.017	0.003	−0.176	0.03	0.134	0.354 **	−0.031	−0.241	−0.017	1					
WA (14%)	0.168	0.753 **	0.092	0.203	0.252 *	−0.321 **	0.784 **	−0.391 **	−0.331 **	−0.166	−0.247 *	−0.255 *	0.148	1				
DDT	0.205	0.321 **	0.091	0.099	0.029	−0.169	0.340 **	−0.143	−0.068	−0.057	−0.182	−0.086	0.402 **	0.392 **	1			
STAB	0.270 *	0.358 **	−0.01	−0.005	−0.088	−0.189	0.132	0.057	0.409 **	0.049	−0.1	0.027	0.538 **	0.279 *	0.422 **	1		
DOS	−0.362 **	−0.595 **	0.084	0.033	0.048	0.258 *	−0.393 **	0.108	−0.231	−0.071	0.089	−0.025	−0.442 **	−0.501 **	−0.398 **	−0.792 **	1	
FQN	0.349 **	0.506 **	−0.048	−0.007	−0.04	−0.19	0.334 **	−0.118	0.234	0.062	−0.116	−0.012	0.489 **	0.511 **	0.516 **	0.905 **	−0.831 **	1

Yellow color highlights positive while orange color highlights negative significant correlation. ** indicates significant correlation at *p* ≤ 0.01. * indicates significant correlation at *p* ≤ 0.05.

## Data Availability

The original contributions presented in the study are included in the article/Supplementary Materials, further inquiries can be directed to the corresponding author.

## Supplementary Materials

The following supporting information can be downloaded at: https://www.mdpi.com/article/10.3390/foods13071125/s1, Table S1. Composition of the identified high molecular weight gluten subunits by SDS-PAGE in the studied wheat cultivars; Table S2. Principal components analysis for rheological traits in wheat genotypes; Figure S1. Principal component analysis (PCA) plot of 55 Indian wheat genotypes and 10 Australian genotypes; Figure S2. Principal component analysis (PCA) biplot of 65 studied wheat genotypes and the measured variables/traits length of arrows indicates the relative size of contribution of the trait.
