# Supplementary material for: Physico-Chemical and Rheological Trait-Based Identification of Indian Wheat Varieties Suitable for Different End-Uses
Source: Foods. 2024 Apr 8;13(7):1125. doi: 10.3390/foods13071125 (PMC11011969; doi:10.3390/foods13071125)
Supplement: Supplementary file 1 [file foods-13-01125-s001.zip › foods-2884394-supplementary.pdf]

Table S1. Composition of the identified high molecular weight gluten subunits by SDS-PAGE in the studied wheat cultivars (*, It's name of HMW-GS protein band).

Code name	Variety	Glu-A1	Glu-B1	Glu-D1	Glu- 1 Score	Code name	Variety	Glu-A1	Glu-B1	Glu-D1	Glu- 1 Score
G-1	Annuello	1	7+8	2+12	8	G-34	HP 1761	2*	17+18	2+12	8
G-2	Barham	1	7+9	2+12	7	G-35	HPW 311	2*	7	5+10	8
G-3	Baxter	1	13+16	2+12	8	G-36	HPW 316	2*	6+8	2+12	6
G-4	Binnu	2*	17+18	2+12	8	G-37	HPW 89	2*	7+8	2+12	8
G-5	C 306	2*	20	2+12	6	G-38	HQW 2	1	7	5+10	8
G-6	Datatine	null	13+16	2+12	6	G-39	HW 2004	2*	13+16	2+12	8
G-7	DBW 14	2*	7+8	2+12	8	G-40	HW 2045	2*	7+8	2+12	8
G-8	DBW 16	2*	7+8	2+12	8	G-41	JANZ	1	7+8	2+12	8
G-9	DBW 17	2*	7	5+10	8	G-42	K 307	2*	17+18	2+12	8
G-10	DBW 39	2*	13+16	5+10	10	G-43	Longreach Orion	2*	7+8	2+12	8
G-11	DL 153-2	null	17+18	5+10	8	G-44	NAPHAL	null	7+8	null	4
G-12	DL 788-2	2*	17+18	2+12	8	G-45	NI 5439	null	7+9	2+12	5
G-13	DPW 621-50	2*	17+18	5+10	10	G-46	NW 1014	2*	7+9	2+12	7
G-14	Drysdale	1	17+18	5+10	10	G-47	NW 2036	1	7+9	5+10	9
G-15	Ega Jitaring	2*	17+18	2+12	8	G-48	PBW 343	2*	7	5+10	8
G-16	Gladius	1	7+8	5+10	10	G-49	PBW 396	2*	7+9	5+10	9
G-17	HD 2329	2*	7+9	2+12	7	G-50	PBW 502	2*	7	5+10	8
G-18	HD 2643	1	7+9	2+12	7	G-51	PBW 550	null	7+9	5+10	9
G-19	HD 2687	2*	7+9	2+12	7	G-52	PBW 590	2*	7+9/7	5+10	9
G-20	HD 2733	null	7+9	5+10	7	G-53	QBP 12-10	null	7+9	2+12	5
G-21	HD 2824	2*	7	5+10	8	G-54	QBP 12-11	null	17+18	2+12	6
G-22	HD 2851	2*	17+18	5+10	10	G-55	QBP 12-8	2*	7+8	2+12	8
G-23	HD 2864	1	7+8	2+12	8	G-56	QBP 12-9	2*	13+16	5+10	10
G-24	HD 2888	null	20	2+12$_2$	4	G-57	QBP 13-10	2*	7	2+12	6
G-25	HD 2894	2*	7+9	2+12	7	G-58	QBP 13-11	2*	8	2+12	6
G-26	HD 2967	2*	17+18	5+10	10	G-59	QBP 13-13	null	7+9	2+12	7
G-27	HD 2985	2*	17+18	5+10	10	G-60	QBP 13-14	null	13+16	5+10	10
G-28	HD 2987	2*	7+8	5+10	10	G-61	RAJ 3765	2*	7+8	2+12	8
G-29	HD 3043	1	13+19	5+10	10	G-62	UP 2425	2*	7+9	2+12	7
G-30	HD 3059	2*	17+18	5+10	10	G-63	WH 1021	2*	7+8	2+12	8
G-31	HDR 77	2*	7+8	2+12	8	G-64	WH 1080	1	13+16	5+10	10
G-32	HI 977	2*	17+18	5+10	10	G-65	WL 711	2*	7+8	2+12	8
G-33	HP 1744	2*	7+9	2+12	7						

Table S2. Principal components analysis for rheological traits in wheat genotypes

Contribution of the variables (%):			
	F1	F2	F3
Glu- 1 Score	5.56	0.11	6.62
Hardness Index (HI)	**10.61**	0.38	20.53
SDS-SV (Flour)	0.35	**18.29**	14.45
Protein (%)	0.36	**23.43**	4.86
Wet Gluten %	2.44	**21.91**	8.73
Dry Gluten %	0.82	**26.92**	2.54
GI	**8.98**	0.76	16.86
W.A. (14%)	**9.85**	2.58	22.00
DDT (minutes)	**8.92**	0.05	0.24
STAB (minutes)	**15.72**	2.70	2.64
DOS (FU)	**17.44**	1.48	0.53
FQN (second)	**18.95**	1.38	0.01

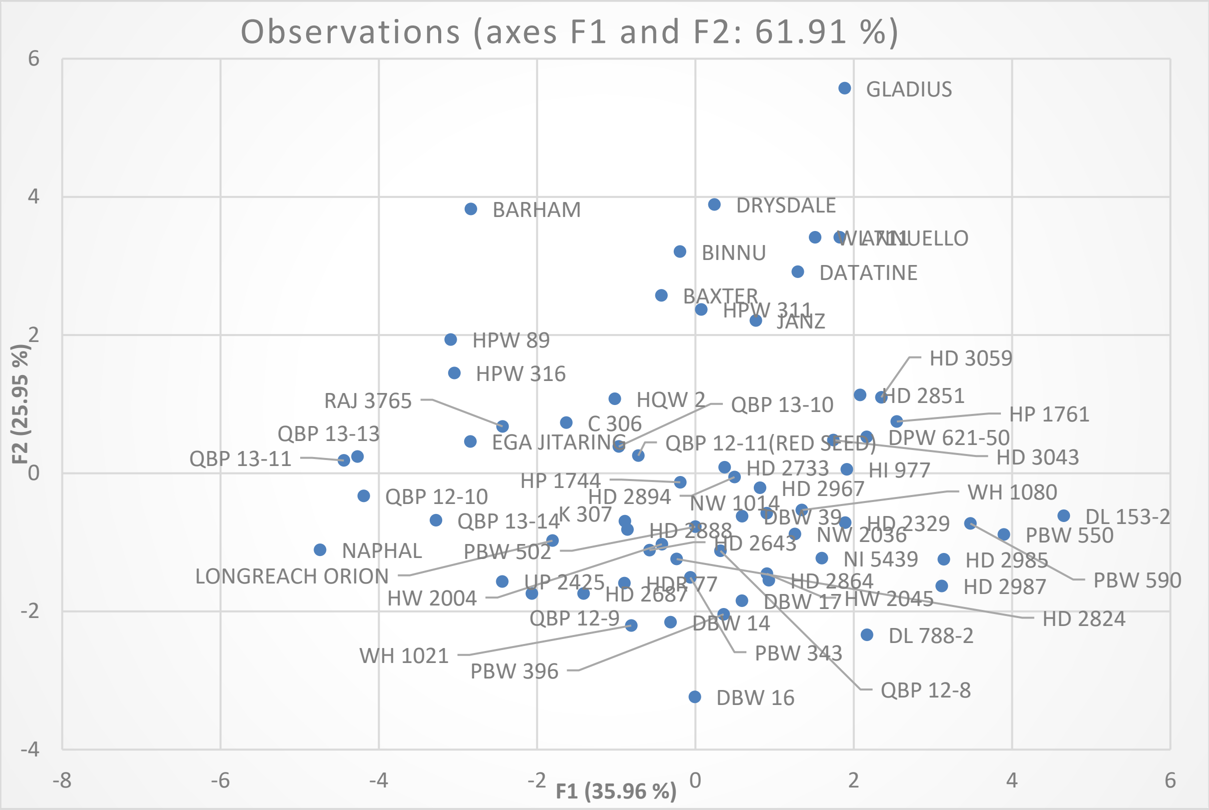

Figure S1. Principal component analysis (PCA) plot of, 55 Indian wheat genotypes and 10 Australian genotypes.

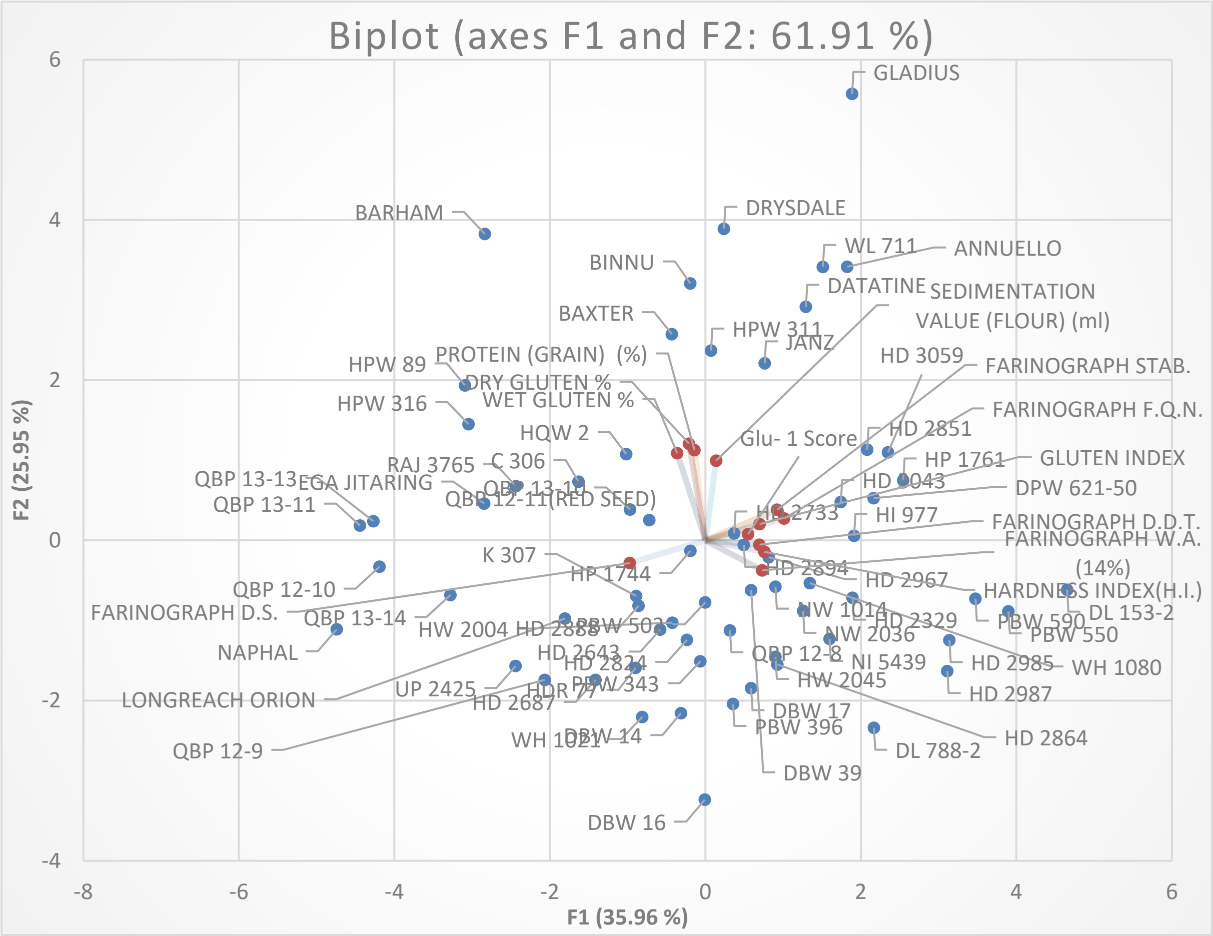

Figure S2. Principal component analysis (PCA) biplot of 65 studied wheat genotypes and the measured variables/traits length of arrows indicates the relative size of contribution of the trait.
